# Heterogenic Final Cell Cycle by Chicken Retinal Lim1 Horizontal Progenitor Cells Leads to Heteroploid Cells with a Remaining Replicated Genome

**DOI:** 10.1371/journal.pone.0059133

**Published:** 2013-03-19

**Authors:** Shahrzad Shirazi Fard, Miguel Jarrin, Henrik Boije, Valerie Fillon, Charlotta All-Eriksson, Finn Hallböök

**Affiliations:** 1 Department of Neuroscience, Uppsala University, Uppsala, Sweden; 2 Laboratoire de Génétique Cellulaire, Institut National de la Recherche Agronomique, Castanet-Tolosan, France; 3 St. Eriks ögonsjukhus, Karolinska Institutet, Stockholm Sweden; Center for Regenerative Therapies Dresden, Germany

## Abstract

Retinal progenitor cells undergo apical mitoses during the process of interkinetic nuclear migration and newly generated post-mitotic neurons migrate to their prospective retinal layer. Whereas this is valid for most types of retinal neurons, chicken horizontal cells are generated by delayed non-apical mitoses from dedicated progenitors. The regulation of such final cell cycle is not well understood and we have studied how Lim1 expressing horizontal progenitor cells (HPCs) exit the cell cycle. We have used markers for S- and G2/M-phase in combination with markers for cell cycle regulators Rb1, cyclin B1, cdc25C and p27Kip1 to characterise the final cell cycle of HPCs. The results show that Lim1+ HPCs are heterogenic with regards to when and during what phase they leave the final cell cycle. Not all horizontal cells were generated by a non-apical (basal) mitosis; instead, the HPCs exhibited three different behaviours during the final cell cycle. Thirty-five percent of the Lim1+ horizontal cells was estimated to be generated by non-apical mitoses. The other horizontal cells were either generated by an interkinetic nuclear migration with an apical mitosis or by a cell cycle with an S-phase that was not followed by any mitosis. Such cells remain with replicated DNA and may be regarded as somatic heteroploids. The observed heterogeneity of the final cell cycle was also seen in the expression of Rb1, cyclin B1, cdc25C and p27Kip1. Phosphorylated Rb1-Ser608 was restricted to the Lim1+ cells that entered S-phase while cyclin B1 and cdc25C were exclusively expressed in HPCs having a basal mitosis. Only HPCs that leave the cell cycle after an apical mitosis expressed p27Kip1. We speculate that the cell cycle heterogeneity with formation of heteroploid cells may present a cellular context that contributes to the suggested propensity of these cells to generate cancer when the retinoblastoma gene is mutated.

## Introduction

Cells of the central nervous system are formed during the process of interkinetic nuclear migration (INM) with S-phases on the basal side followed by apical mitoses [1]–[3]. Once the cells undergo the terminal/neurogenic mitosis they migrate out and withdraw from the cell cycle [4]. Cortical progenitors either undergo terminal mitosis at the apical surface of the neuroepithelium or they initiate differentiation and undergo a delayed terminal mitosis in the subventricular zone during migration [5]–[7]. Such delayed non-apical terminal mitosis serves a mechanism for expansion of a particular cell type. Newly generated post-mitotic cortical cells then continue to migrate to their final destinations in the cerebral cortex [8].

The retina consists of neurons that undergo terminal mitosis on the apical side [9] and post-mitotic cells migrate to their prospective retinal layer. This is valid for most of the five retinal neuronal classes but not for horizontal cells (HCs), which can be generated by non-apical mitoses. In the chicken retina these terminal mitoses occur on the basal side [10], [11] and in the zebrafish retina in the HC layer [12]. Before the terminal mitosis, horizontal progenitor cells (HPCs) express HC-characteristic markers such as Ptf1a, Prox1, Lim1, Isl1 and Cx55.5. The HPCs are thereby able to remain in the cell cycle and perform an additional mitosis after initiating differentiation [10]. The expression of differentiation markers before the terminal mitosis resembles that of the cortical neurons, which initiates differentiation and migration before the neurogenic non-apical mitosis [6]. Another similarity between migrating HPCs and cortical progenitors is the expression of doublecortin [13], [14].

Chicken and most vertebrate HCs can be divided in two groups based on the expression of the transcription factors Lim1 or Isl1 [11], [15], [16]. In chicken the Lim1 positive (+) HCs (axon bearing HCs, H1 subtype) constitute 50% of all HCs [11], [17] and are generated one day before the Isl1+ HCs (axon-less HCs, H2, H3 subtypes). We focused on the Lim1+ H1 HCs because they are a well-demarcated population and have the non-apical terminal mitoses. Lim1 is expressed exclusively in H1 HCs during their final cell cycle and in mature HCs [10], [11], [15], [16], [18]. Previous work based on [^3^H]-dT incorporation, indicated that the Lim1+ HPCs go through a final S-phase at st19–25. It was also demonstrated that the HPCs migrate to the basal side of the retina where they accumulate and divide before migrating back to the prospective horizontal layer [10], [11]. The final S-phase and the cell cycle behaviour of HPCs differs from the INM seen by other retinal cells and thus it was hypothesised that the strict association between nuclear position and cell cycle phase, as seen during INM, may be de-regulated during the final cell cycle of HPCs.

In this work we studied the final cell cycle of HPCs, with regard to the S-, G2-phases and the observed basal mitosis [11]. We used markers for S- and G2/M-phase in combination with markers for cell cycle regulators, Rb1 (G1-phase), cyclin B1, cdc25C (late G2/M-phase) and p27Kip1 (post-mitotic phase), to characterise the final cell cycle of HPCs. Unlike what was expected, the results showed that the final cell cycle of Lim1+ HPCs is heterogenic. The results indicate that Lim1+ HPCs exhibit at least three different final cell cycle behaviours. We found that approximately 35% of the Lim1+ HPCs underwent a basal mitosis. The rest of the Lim1+ HPCs consisted of cells that went through S-phase, which was not followed by mitosis and of cells that seemed to have their last cell cycle during an INM. The HCs were therefore divided into three categories based on their behaviour during the final cell cycle: First, Lim1+ HPCs that exit the cell cycle after a normal INM and migrate as post-mitotic cells to the basal side of the retina; second, Lim1+ HPCs that undergo S-phase while migrating to the basal side. Their S-phase is not followed by mitosis and these cells remain with a replicated genome and may be regarded as somatic heteroploids. The third type of behaviour is cells that undergo S-phase while migrating to the basal side followed by a terminal basal mitosis before settling in their final laminar position. This third kind of behaviour was described previously [10], [11]. Rb1, cyclin B1, cdc25C and p27Kip1 were expressed in a heterogenic pattern; cells with behaviour “two” and “three”, having a delayed final cell cycle, expressed hyperphosphorylated Rb1 during S-phase. Cyclin B1 and cdc25C were only seen in HPCs classified as having behaviour “three” during their basal mitosis. p27Kip1 was expressed by the HPCs that leave the cell cycle after an apical mitosis (behaviour “one”).

The heterogeneity in the final cell cycle of HPCs is of interest since HCs have recently been shown to re-enter the cell cycle and form a clonally HC-derived metastatic cancer in a mouse model for retinoblastoma [19]. It may therefore be important to understand the variability in the regulation of the final cell cycle in HPCs.

## Methods

### Ethics Statement

This study was carried out in accordance with the recommendations in the Guide for the Care and Use of Laboratory Animals of the Association for research in vision and ophthalmology. The protocol was approved by the Committee on the Ethics of Animal Experiments by Uppsala djurförsöksetiskanämnd (Permit number C14/9).

### Animals

Fertilized White Leghorn eggs (*Gallus gallus*) were obtained from Ova Production AB (Västerås, Sweden) and incubated at 38°C in a humidified incubator (Maino, Oltrona di San Mamette, Italy). Embryos were staged according to Hamburger and Hamilton stages (st) [20] and denoted either as st or the corresponding embryonic age in days (E).

### Immunohistochemistry

Immunohistochemistry was performed as described previously [18]. Briefly, tissues were fixed in 4% paraformaldehyde in PBS for 15 min, 1 h or 3 h depending on the primary antibody, followed by 10 min wash with PBS and cryoprotection in 30% sucrose for 3 h before being frozen in OCT (Sakura). Tissues were cryosectioned and collected on Superfrost Plus glasses, followed by incubation in blocking solution (PBS containing 1% FCS and 0.1% Triton X-100, 0.02% Thimersal) for 30 min. Primary antibodies were allowed to react over night at 4°C, and secondary antibodies for 2 h at room temperature. Primary and secondary antibodies were diluted in blocking solution. The slides were coversliped with ProLonged Gold with DAPI to visualize nuclei. The following antibodies were used; the transcription factor Lim1/2 (1∶20, mouse, 4F2-s, Developmental studies hybridoma bank (DSHB), Prox1 (1∶4000, rabbit, AB5475, Chemicon), Cells in late G2 or M-phase were identified using an antibody to PhosphoHistone H3 (PH3) (1∶4000, rabbit, 06–570, Millipore; 1∶400, goat, sc-12927, Santa Cruz), BrdU (1∶100, rat, OBT0030F, AbDserotec), Phosphorylated Rb1 s608 (1∶4000, rabbit, ab60025, abcam), Caspase-3, cleaved (1–4000, rabbit, #9661, Cell Signaling), p27Kip1 (1∶200, mouse #610241, BD Transduction Labs), cdc25C (1∶1000, rabbit, ab47329, Abcam), secondary antibodies were obtained from Abcam or Invitrogen. Samples were viewed and analysed using a Zeiss Axioplan 2 microscope equipped with an AxioCam C camera and Axiovision software or using a Zeiss LSM 510 confocal microscope equipped with LSM 510 imaging software (v4.2). Images were formatted, resized, enhanced by brightness and contrast, and arranged using Axiovision or Imaris software (v7.4, Bitplane Scientific Software AG, Zürich, Switzerland) and Adobe Photoshop CS4.

### EdU Injections

The thymidine analogue 5-ethynyl-20-deoxyuridine (EdU) [21] (Click iT EdU imaging kit C10084, Invitrogen, USA) was used to visualize cells in S-phase [22]. Injections were done in either the embryonic eye or yolk sac to produce a pulse of EdU or a continuous exposure adequate to label proliferating cells thereafter. Single eyes of st24, st27 and st29 embryos were injected with 1.25 µg EdU dissolved in 1 µl PBS. The eyes were collected after 15 min, 1 h or 3 h. Yolk sac injections of st26–31 embryos were performed with 50 µg EdU. The eyes were collected after 6 h or at st33 or st42. The yolk sac approach can be compared with the cumulative labelling by [^3^H]-dT of cells entering the cell cycle [23]. All of the eyes were fixed, frozen, sectioned and stained with immunohistochemistry and the EdU labelling was performed according to the manufacturer’s protocol. The duration of the EdU exposure after embryonic eye or yolk injections was analysed.1.25 µg EdU was injected (T0) into the eyes of st24 embryos immediately followed by injection of 5 µg BrdU (T0) or after 2 h (T2) or 4 h (T4). Samples were collected 6 h after the initial EdU injection and the fraction of EdU, BrdU co-labelled cells was analysed and calculated. For the yolk experiment, a single 50 µg EdU yolk injection in st26 embryos was compared to two repeated EdU injections 48 h in between (at st26 and st30). The samples were collected at st33, approximately 4 days after the initial EdU injection, and the number of EdU labelled Lim1+ cells was analysed in the animals with a single compared with repeated injections.

### Cell Counts

Cell counts were based on 4 different animals if otherwise is not stated. Minimum 4 sections from each animal were counted. All eyes were oriented based on the pecten and sectioned identically. For all stage-specific cell counting, only the central part of the retina was analysed to avoid bias imposed by the temporal and centro-peripherial aspects of retinal development. When counting ventricular (apical) and vitreal (basal) cells the retina was divided in 3 areas and the inner third was considered as basal. The mean (+/− SD) for each combination of labelling and stage was calculated and the data were analysed and presented in GraphPad Prism (v3.02, GraphPad software Inc.). Statistical analysis; Analysis of variance (one-way ANOVA) followed by Tukey's multiple comparison post-hoc test and p-values<0.05 were considered significant.

### In ovo Electroporation with Cyclin B1-GFP Expression Vector

A cyclin B1-GFP vector (G2M cell cycle phase marker pCORON4004-CCEGFP expression vector, NIF2034, Amersham Biosciences) was used to visualize cyclin B1 in HPCs. Cell cycle specific expression was driven by a human *CCNB1* promoter, intracellular localisation as well as the cyclic degradation of cyclin B1 protein was investigated and confirmed in vitro by Lipofectamine™ 2000 (11668–027, Invitrogen) treatment of chicken DF1 fibroblasts with the construct followed by incubation for 48 h. Embryonic st22 eyes were injected subretinally with the pCORON4004-CCEGFP cyclin B1-GFP vector (5 µg/µl) and electroporation was carried out using a BTX ECM830 electroporator delivering five 50 ms pulses of 23V. The eyes were collected at st25 and st27 and analysed with immunohistochemistry.

### Fluorescence-activated Cell Sorting (FACS) Analysis

Eyes were collected from chicken embryos just before hatch (P0). P0 retinas were used to avoid late dividing Prox1 bipolar cells. Cornea, lens and vitreous were removed and the retina with pigment epithelium still attached to the sclera was incubated in PBS (Ca^2+^, Mg^2+^-free), 2 mM EDTA, 5.5 mM glucose and 10 mM Hepes for 15 min on ice to facilitate the removal of the pigment epithelial cells. The central region of the neural retina was then dissected from 4 retinas and all pigment epithelial cells were removed. The tissue was incubated in 0.5 mg/ml trypsin in PBS for 7 min at 37°C. After removal of the trypsin solution, a single cell suspension was obtained by gently trituration of the retina in 0.5 ml of FBS. The cell suspension was washed once in PBS and fixed with 70% ethanol for 10 min and passed through a 40 µm cell strainer (Falcon, BD Biosciences, San Jose, CA). Cells were again washed with PBS and incubated for 60 min at room temperature in blocking solution (0.1% Triton X-100, 0.02% Thimersal and 1% FCS in PBS). After blocking, cells were incubated with the Prox1 antibody diluted in blocking solution (1∶4000, rabbit, AB5475, Chemicon) and incubated overnight at 4°C with gentle agitation. Cells were washed three times with PBS and incubated with an anti-rabbit Alexa 488 (Invitrogen) diluted 1/1000 in blocking solution. Finally, the cells were washed three times in PBS. Single cells suspensions were incubated 30 min with 25 µg/ml ribonuclease A (Sigma, R4875), followed by 25 µg/mL propidium iodide (PI, Sigma, P4864) for 30 min at room temperature.

Negative, positive and secondary antibody controls were performed. Cell analysis was carried out using Guava EasyCyte 8 cell analyser (Millipore, Billerica, MA, USA). Aggregates were excluded from the analysis and values from primary antibody positive and primary antibody-negative cells were gated using the maximal intensity level of cells incubated with the secondary antibody alone as a threshold.

### Measurement of Lim1+ Nuclei Volumes

The nucleus volume of st42 Lim1+ HCs was determined using immunohistochemistry, confocal microscopy and 3D analysis. Z-stacks projections were used to generate 3D models of the HC layer. Lim1 staining was used to generate an iso surface by use of the Imaris software, Vantage module and the volume of the Lim1+ nuclei was calculated using default value settings.

### Fluorescence in situ Hybridisation (FISH) on Tissue Cryosections

Interphase nucleus FISH was performed on cryosections with directly labelled bacterial artificial chromosome probes in combination with immunohistochemistry for Prox1 or Lim1. Immunohistochemistry on cryosections in combination with FISH has been described previously [24]. FISH was performed with a bacterial artificial chromosome probe for chromosome Z on sections of female chickens in order to have a single copy of the probed region. The Z probe produces a single spot in female and two spots in male cells. After the initial immunohistochemistry, the sections were fixed 20 min in 2% paraformaldehyde prior to FISH. The Z BAC clone (B6A6) [25] covers approximately 25% of the distal part of Z chromosome and was labelled with Alexa Fluor 568-5-dUTP (ChromaTide, Molecular Probes) by random priming using the Bioprim Kit (Invitrogen, USA). The probe was purified using a spin column G50 Illustra (Amersham Biosciences), ethanol precipitated and resuspended in 50% formamide hybridization buffer. Slides were pre-incubated 5 min in 10 mM sodium citrate at room temperature, 5 min in 10 mM sodium citrate at 80°C, cooled down at room temperature, than incubated 5 min in 2XSSC, 1 h in 50% formamide in 2XSSC and briefly rinsed with 2XSSC. The probe was loaded on the slide, coversliped and sealed with rubber cement. The slides with probe were pre-warmed at 45°C for 1 h, denatured at 80°C for 5 min and hybridized for 3 days at 37°C in a Hybridizer (Dako, Glostrup, Denmark). After hybridization, the slides were washed 3×15 min in 2XSSC at 37°C, 2×5 min in 0,1XSSC at 60°C and 2 min in 2XSSC at room temperature. Tissue sections were counter stained with DAPI and coversliped in Vectashield (Vector, Burlingame, CA, USA). The slides were analysed by confocal microscopy using Z-stack scans. The pinehole was optimized for each laser wavelength (488 nm and 568 nm) in order to get the same optical slice for each wavelength. The Z-stacks were projected as a 3D model using the Imaris software.

### Determination of the Sex of the Embryos

The sex of the embryos was determined by analysing the expression of W-linked protein kinase C inhibitor-8 (WPKCI-8) mRNA by using quantitative reverse transcriptase polymerase chain reaction (qRT-PCR) as described in [26], [27]. Briefly, complementary DNA was made from total RNA and two-step qPCR were performed using the iQ SYBR Green supermix (Bio-Rad Laboratories, Inc., CA, USA) and MyiQ Single-Colour Real-Time PCR Detection System (Bio-Rad Laboratories, Inc.). Primers were designed with Primer Express 1.5 (Applied Biosystems, Carlsbad, CA, USA) software; WPKCI-8 mRNA identifier AB026677.1; forward primer AGATTGTGGCGCACCTCTTC, reverse primer CACTTCTCGCCAACAATCATCA. Male tissue does not express the gene.

## Results

### Not All Lim1+ HPCs go through S-phase as they Accumulate on the Basal Side of the Retina

To localise Lim1+ nuclei undergoing S-phase in relation to their position in the retina, the tymidine analogue EdU was injected into eyes of st24 embryos. The number of Lim1+ HPCs that go through their final S-phase peaks at st24 [11], making their detection more easy at this stage. The eyes were collected 15 min, 1 h or 3 h after EdU injection and analysed for expression of Lim1, PH3 (marker for late G2 and M-phase [28]) and incorporation of EdU. The 15 min pulse displayed the position of the S-phase nuclei and the increasing lengths of EdU pulses allowed us to monitor accumulation of EdU, Lim1 double-positive cells on either side of the retina. The times were sufficient short not to catch two subsequent S-phases by the same cell.

**Figure 1:**
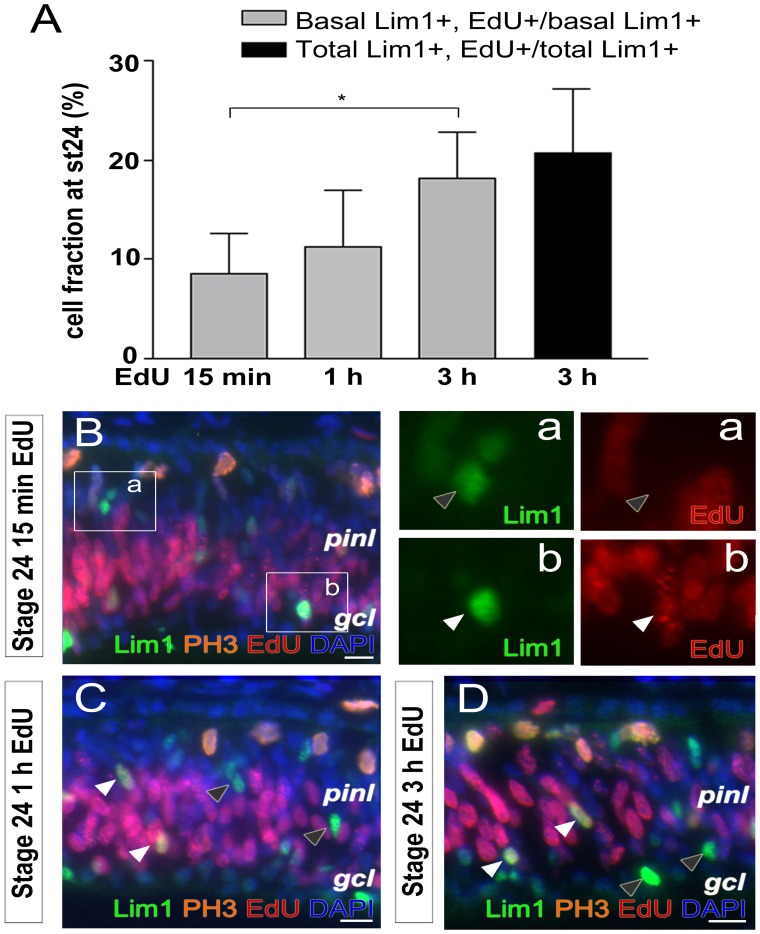
Lim1+ HPCs have an S-phase while migrating to the basal side . (A) Fraction of Lim1+ cells at st24 that have incorporated EdU at different pulse lengths. The basal Lim1+ cells (light grey bars) and total Lim1+ cells, basal and migrating (dark grey bar) were counted. (B) Lim1, EdU and PH3 immunolabelling after (B) 15 min, (C) 1 h and (D) a 3 h-EdU pulse in st24 retinas. White arrow head; double-positive cell, grey arrow head; single-positive cell, gcl; ganglion cell layer, pinl; prospective inner nuclear layer, st; Hamburger and Hamilton stages. One-way ANOVA followed by Tukey's multiple comparison post-hoc test, *p<0.05, n>4 treated eyes, 4 sections per eye. Scale bar is 10 µm.

The 15 min EdU pulse gave some spotted, partially labelled nuclei in the prospective inner nuclear layer (INL) in addition to the cells on the basal side. We focused on the basal distinctly Lim1+ cells and after 15 min EdU pulse 8.5±4.1% of the cells were EdU+ (Fig. 1A, n = 4). EdU exposure for 1 h or 3 h resulted in proportionally more Lim1, EdU double-positive cells located on the basal side (11.3±5.6% and 18.1±4.7% respectively) (Fig. 1A, n = 4). Lim1, EdU double-positive (Fig. 1B–D, white arrow heads) and Lim1+, EdU negative cells (Fig. 1B–D, grey arrow heads) were seen both in the prospective INL and on the basal side. Lim1, EdU double-positive cells were not seen on the apical side of the retina. The increase of Lim1, EdU double-positive cells on the basal side and absence on the apical side suggested that the Lim1+ cells accumulated on the basal side after S-phase. The percentage of EdU+ cells of the total number of Lim1+ cells at st24 was 20.7±6.4% (basal and prospective INL cells) (Fig. 1A, n = 4).

The presence of both Lim1, EdU double-positive and Lim1+, EdU negative cells in the prospective INL and on the basal side suggested that some but not all Lim1+ cells go through S-phase during the 3 h exposure period. This is consistent with that Lim1+ HCs exit the cell cycle in different ways as they migrate towards the basal side of the retina. We therefore categorized HCs, based on their hypothesised final cell cycle behaviours, in two groups; behaviour “one” and “two”. Cells with behaviour “one” migrates as post mitotic cells and cells with behaviour “two” go through an S-phase.

The st24 retinas that had been exposed for EdU were stained for Lim1, EdU and PH3 in order to visualise mitoses. PH3, EdU double-positive cells were seen on the apical side (Fig. 1D) but neither apical nor basal Lim1, PH3 double-positive cells were observed (Fig. 1B–D). This showed that the Lim1+ cells that go through an S-phase at st24 do not enter late G2-phase or mitosis within 3 h after their S-phase.

An experiment was performed to investigate the duration of the EdU exposure after eye injection. BrdU was injected simultaneously, 2 h or 4 h after an EdU injection and co-labelling of EdU and BrdU was analysed. EdU, BrdU double-positive cells were seen in the retina with the simultaneous BrdU injection and in retinas injected with BrdU 2 h after the EdU injection. The number of EdU, BrdU double-positive cells had declined in eyes with a BrdU injection 4 h after EdU injection showing that the duration of the EdU pulse lasted for more than 2 h but was shorter than 4 h (Supporting Fig. S1A–D, n = 2).

### Half of all Lim1+ HPCs Undergo S-phase after st26

**Figure 2:**
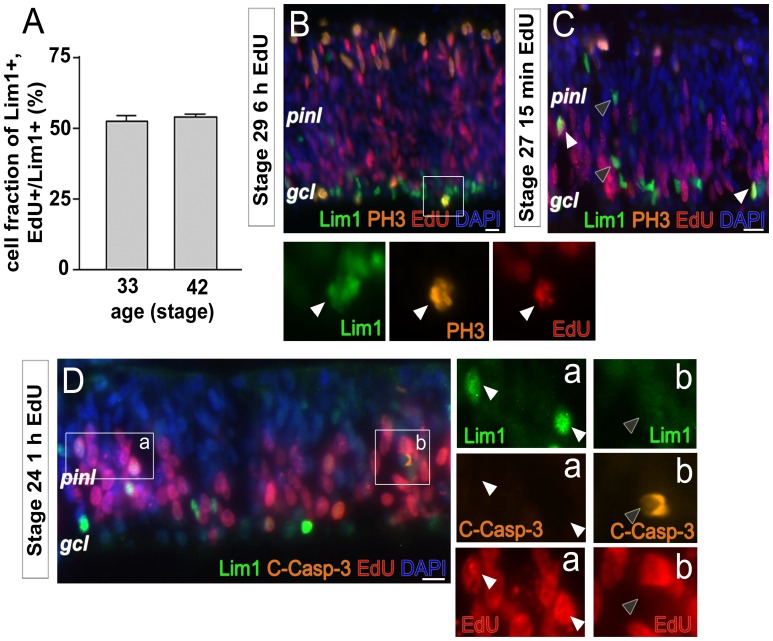
Lim1+ HPCs basal mitoses are preceded by an S-phase. (A) Bargraph showing the fraction of Lim1+ cells at st33 and st42 that have incorporated EdU after a yolk injection. The non-apical terminal mitoses of HCs, and only HCs, occur on the basal side by the ganglion cell layer [10]. Fluorescence micrograph showing Lim1, PH3 and EdU after a 6 h-EdU pulse at st29 and (B) after a 15 min-EdU pulse at st27. (D) Lim1, EdU and cleaved caspase-3 (C-Casp-3) immunoreactivity after a 1 h-EdU pulse. White arrow head; double- or triple-positive cell, grey arrow head; single-positive cell, gcl; ganglion cell layer, pinl; prospective inner nuclear layer, st; Hamburger and Hamilton stages. Scale bar is 10 µm.

We have previously demonstrated that many Lim1+ HC progenitors go through an S-phase at early stages (st19–25) of HC development but that HCs are formed by a basal mitosis that occurs at st26–31. However, Lim1+ cells with an S-phase after st26 can be observed [10], [11]. To determine the fraction of Lim1+ HPCs having an S-phase after st26, EdU was injected in the yolk of st26 embryos. EdU injected in the yolk serves as a deposit and will label all S-phases thereafter. The fraction of all Lim1+ cells in the HC layer (central part of the retina) that were EdU+ was analysed and counted at st33 and st42. At st33 the Lim1+ HCs have arrived to their final laminar position and at st42 the HCs have settled in the HC layer and both time points represent times well after when Lim1+ HCs are considered to be post-mitotic. At st33 52.7±2% and at st42 54.0±2% of the Lim1+ cells were EdU positive, indicating that half of all Lim1+ HPCs have had an S-phase after st26 (2465 cells counted, n = 4, bar graph in Fig. 2A).

An experiment was performed where a single EdU yolk injection was compared to two repeated injections into the yolk at st26 and at st30. The fraction of Lim1, EdU double-positive cells in the central part of the retina was similar in the single injection as in the retina with repeated injections when analysed at st33 (single injection 53.4±7% and double injection 54.5±6% of the Lim1+ cells were EdU positive, 1024 cells counted, n = 2, Supporting Fig. S1E–G). This shows that a single injection at st26 lasted over the period of HC genesis.

### All Basal Mitoses are Preceded by an S-phase within 6 h but not all HCs are Formed by these Mitoses

The fraction of Lim1+ HPCs having an S-phase after st26 was higher than expected and raised the question if all Lim1+ HPCs with a delayed basal mitosis undergo S-phase after st26. These HPC mitoses (basal Lim1, PH3 double-positive cells) can be observed from st26 to st31 with a peak at st29–30 [10]. The basal mitoses were therefore investigated by injecting EdU in the yolk at every stage from st26–31 with analysis after 6 h. The duration was chosen to exclude cells that have undergone two S-phases or cells that have an extended G2-phase [29]. Triple-labelling for Lim1, EdU and PH3 was performed with focus on basal PH3+ cells (Fig. 2B). Analysis and counting at all stages from st26 to st31 showed that more than 99% of the Lim1, PH3 double-positive cells were EdU positive (573 cells counted, n = 4/stage). The analysed ages include the vast majority of the basal mitoses and the result showed that all basal Lim1+ HPC mitoses have S-phase and mitosis within 6 h. This result suggests that Lim1+ cells with an S-phase followed by a basal mitosis within 6 h, represent a third kind of final cell cycle behaviour that occurs late (S-phase after st26) during HC formation.

We repeated the same experiment at st27 and st29, which we had performed at st24. Eyes were injected with EdU and analysed 15 min, 1 h or 3 h after the injection. At st27 Lim1, EdU double-positive and Lim1+, EdU negative cells were observed in the prospective INL (Fig. 2C). This result suggests that some of the Lim1+ HPCs located in the prospective INL undergo an S-phase, whereas others do not. When we analysed co-labelling of Lim1 and PH3, no apical Lim1, PH3 double-positive cells were observed, indicating that these S-phases were not followed by an apical mitosis. The same detailed analysis was performed at st29; both Lim1, EdU double-positive and Lim1+, EdU negative cells were observed in the prospective INL. No Lim1, PH3 double-positive cells were seen on the apical side of the retina (data not shown). To summarise, the results suggest that there are Lim1+ cells at both st27 and st29 that do not go through S-phase within the 3 h analysis time and that these cells exhibit a similar behaviour denoted as behaviour “one” in st24 retina.

We wanted to investigate if the Lim1+ cells undergo apoptosis and therefore analysed cleaved caspase-3 (C-Casp-3) immunoreactivity. We did not find any Lim1 single positive or Lim1, EdU double positive cell that were C-Casp-3+. As an internal control for the analysis, C-Casp-3+, Lim1 negative cells were observed throughout the retina (Fig. 2D, grey arrow head). The result is consistent with that developing HCs do not undergo naturally occurring cell death or apoptosis [10], [11], [30].

### No Late Ectopic HPC Mitoses in the HC Layer

Our results suggested that some Lim1+ HPCs have an S-phase at st19–25 before or while accumulating on the basal side and that this is not directly followed by mitosis (either basal or apical). In zebrafish, HPCs enter the HC layer and undergo delayed non-apical mitosis at the final HC laminar location [12]. We investigated retinas of st33 (E8) – st44 (E18) for mitoses in the HC layer, by staining for Lim1 and PH3. At st33 the HCs have reached their final laminar position [11], [31]. No Lim1, PH3 double-positive cells were seen in the HC layer (Supporting Fig. S1H, 0 PH3+ of 2532 Lim1+ cells inspected in st33–44, n = 2/stage, all stages between st33 to 44 were inspected), showing that chicken HCs (at least up to st44) do not divide in the HC layer.

### The Prox1+ HC Population Contains Cells with Increased DNA Content

**Figure 3:**
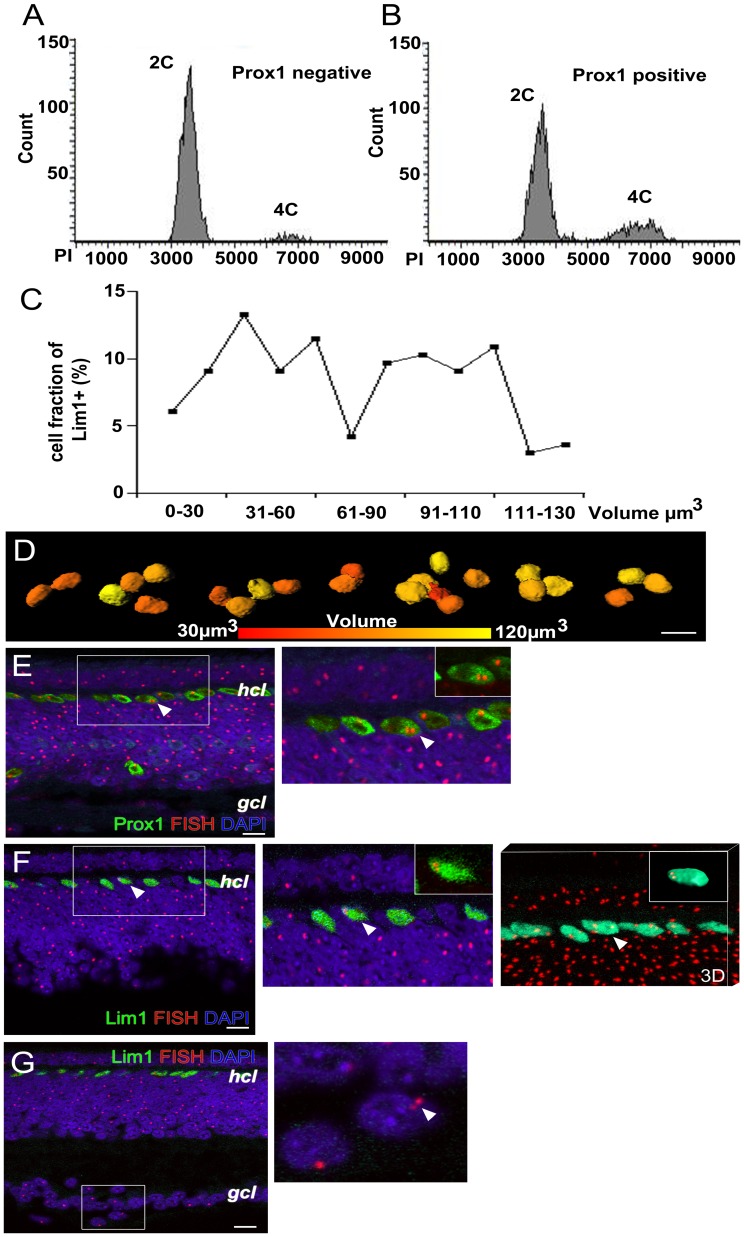
Detection of remaining replicated cells by FACS, nucleus volume and Z-chromosome FISH analysis of interphase nuclei. P0 chicken retinas were incubated with anti-Prox1 antibody, counterstained with PI and analyzed by using flow cytometry. (A) Prox1 negative cells only show a small population with increased DNA content (4C, 6.3±2.2%) and (B) a subset of Prox1 + cells with 4C DNA content (28.9±1.3%). (C) Determination of the volume of st42 Lim1+ nuclei (D) illustration of the differences in the volume. Prox1 or Lim1 immunohistochemistry in combination with Z-chromosome FISH analysis of interphase nuclei in cryosections. The FISH analysis produces one red fluorescence signal (spot) per diploid female cell. Confocal section through the central region of a female st42 retina (E) Prox1+ HC nuclei and (F) Lim1+ HC nuclei and the HC layer (hcl) are depicted. The framed area is both magnified and is shown as a 3D reconstruction of the confocal stack. Insets are one cell having a double fluorescence signal (two spots). The Lim1+ cell is also shown as an animated rotation of the 3D reconstruction in the supporting Fig. S3. (G) Confocal section of st42 retina depicting a cell positioned in the ganglion cell layer with two red spots indicating a replicated genome. Inset is a magnification of the cell. White arrow head; cells with double-spots. gcl; ganglion cell layer, hcl; horizontal cell layer. Scale bar is 10 µm.

Our results suggested that some of the HPCs initiate S-phase without entering a subsequent M-phase. Such cells should contain more DNA (4C) than a normal cell (2C). We used fluorescence-activated cell sorting analysis (FACS, flow cytometry) to analyse the DNA content in HCs from the P0 retina. Lim1 antibodies were incompatible with the flow cytometry and instead we used Prox1 as a HC marker. Prox1 is expressed in all HC and some bipolar and amacrine cells [11], [31]. At P0 72,0±3,8% of the Prox1+ cells were located in the HC layer (n = 2, Supporting Fig. S2A–B). The DNA content in Prox1+ cells was analyzed by propidium iodide DNA binding. Cells with a higher DNA content were more abundant in the Prox1+ population than in the Prox1 negative population. 28.9±1.3% of the Prox1+ and 6.3±2.2% (n  = 2) of the Prox1 negative population had an increased DNA content (Fig. 3A–B). If all Prox1+ cells with higher DNA content were HCs, they would at most constitute 40% of the HC population.

### Volume of Lim1+ Nuclei in the HC Layer

Nuclear volume has been used to study DNA content and ploidy [32]–[34]. We analysed Lim1 immunohistochemistry stained st42 retinas with confocal laser scanning microscopy. Z-stacks were used for 3D modeling of the Lim1+ nuclei in the HC layer and for determining nuclei volumes. A homogenous diploid DNA content can be expected to give a distinct volume within a specific cell type [34]. 150 nuclei were analysed and the volume range in the analysis was from 30–130 µm^3^. The volume distribution was rather broad and gave a bimodal pattern with a minimum at 69–90 (70) µm^3^ rather than one dominating nuclear volume around the mean (Fig. 3C). The result indicated a heterogenic DNA content. If 70 µm^3^ is considered to be a breakpoint between small and large nuclei, 81 (54%) could be classified as small and 69 (46%) as large. The position of large and small Lim1+ nuclei in the HC layer was alternating (Fig. 3D).

### Z-chromosome FISH Analysis of HCs

We used a combination of Prox1 or Lim1 immunohistochemistry with chromosome FISH on interphase nuclei in cryosections of st33 and st42 retinas to look for cells with additional chromatids. Cells that undergo S-phase but not mitosis should have the double number of chromatids. We used a Z-chromosome BAC probe that recognises the single Z-chromosome in female nuclei and produces one fluorescent spot per nucleus. We hypothesised that a nucleus with replicated DNA would have a two-spot pattern. The immune-FISH results were analysed by confocal microscopy with 3D reconstruction of Z-stacks (Fig. 3E–G, Supporting Fig. S3 and S4C–D). Metaphase chromosome FISH showed that the probe hybridized to approximately 25% of one of the Z-chromosome arms. Analysis of interphase nuclei produced double spots in cultured male chicken embryonic fibroblasts and a single spot in female cells. The spots were often located in the periphery of the nucleus (Supporting Fig. S4A and B). Both Prox1 and Lim1 immunostained female st42 retinas were analysed; Two Prox1+ nuclei with clear double spots were observed out of 100 Prox1+ nuclei inspected. No Prox1+ nuclei with double-spots could be found outside the HC layer (100 Prox1+ nuclei inspected). Two Lim1+ nuclei with clear double spots and three nuclei with elongated spots were observed out of 300 inspected Lim1+ nuclei (Fig. 3E–F). In order to learn what a replicated cell may look like we inspected the far periphery of st33 retinas where mitotic Lim1+ cells accompanied with cells in G2-phase with a replicated genome can be observed. No clear two-spotted nuclei could be seen. Instead, elongated spots were seen in Lim1+ cells (Supporting Fig. S4C and D). The average size of one spot was 0.88±0.15 µm (100 spots measured), while the elongated spots were >1.5 µm. We interpreted the result as if the elongated spot represented two sister Z-chromatids and that replicated chromatids can be hard to visualise because of their proximal position. Cells with somatic tetraploidy have recently been described in the ganglion cell layer [32] and we observed cells with two spots in the ganglion cell layer (Fig. 3G).

### Heterogenic Expression of Rb1 and p27Kip1 in HPCs

**Figure 4:**
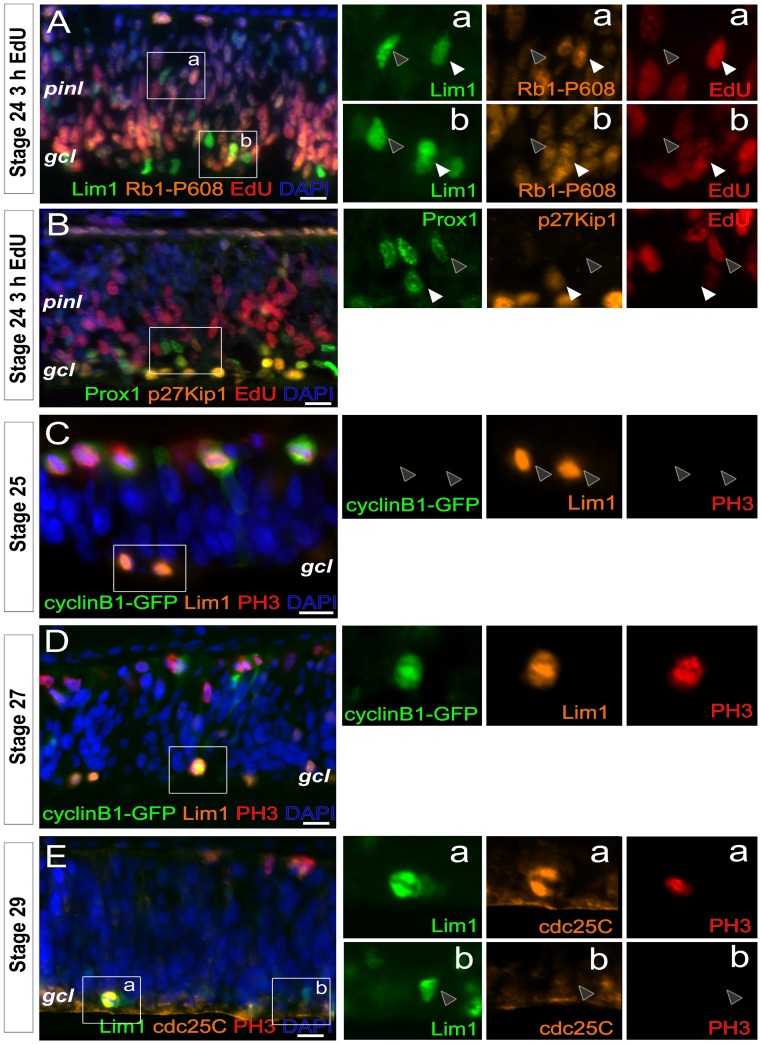
Expression of Rb1-P608, p27Kip1, cyclin B1 and cdc25C in HPCs. (A) Fluorescence micropgraphs with Lim1, Rb1-P608 and EdU immunoreactivity in st24 retina after a 3 h-EdU pulse, (B) Prox1, p27Kip1 and EdU immunoreactivity in st24 retina after a 3 h-EdU pulse. Fluorescence micrographs with cyclin B1-GFP, Lim1 and PH3 in (C) st25 and (D) st27 retina. Note that Lim1+ cells are negative for cyclin B1 and PH3 (grey arrow heads) at st25 but positive for cyclin B1 and PH3 at st27. (E) Fluorescence micrographs with Lim1, cdc25C and PH3 immunoreactivity in st29 retina. White arrow head; double- or triple-positive cell, grey arrow head; single-positive cell, gcl; ganglion cell layer, pinl; prospective inner nuclear layer, st; Hamburger and Hamilton stages. Scale bar is 10 µm.

The cell cycle progression in retinal progenitors is regulated by the expression and phosphorylation of Rb1 and the cell cycle exit by p27Kip1 [35]–[37]. The Rb1 protein is hyperphosphorylated during G1-phase leading to the release and activation of E2f family members and thereby entry into S-phase. One of the crucial phosphorylation sites on pRb1 is S^608^ [38] and we used a specific antibody to detect phospho-S^608^-Rb1 (Rb1-P608). Cells expressing Rb1-P608 are expected to be in late G1-phase or S-phase [38]. We analysed st24 retinas 3 h after EdU injection and triple-stained for Lim1, EdU and Rb1-P608. Lim1, EdU double-positive cells were all Rb1-P608 positive (Fig. 4A, white arrow heads, more than 200 cells inspected, n = 4). The Lim1+, EdU negative cells were Rb1-P608 negative (Fig. 4A, grey arrow heads, more than 200 cells inspected, n = 4). The same analysis was performed at st27 with similar results. The results were consistent with the hypothesis that a proportion of the Lim1+ cells are post-mitotic.

Next we analysed the expression of p27Kip1. The Lim1 and p27Kip1 antibodies are both made in mouse and we had to use a rabbit Prox1-antibody as a marker for HCs. The transcription factor Prox1 is expressed in differentiating Lim1+ HCs [31], [39]. We analysed st24 retinas 3 h after EdU injection and triple-stained for Prox1, EdU and p27Kip1. The result showed that most of the p27Kip1+ cells were located on the basal side of the retina and those cells were Prox1, EdU double-negative post-mitotic retinal ganglion cells (data supported by ganglion cell specific markers and not shown). In addition, we found Prox1, p27Kip1 double-positive cells (Fig. 4B, white arrow head), which consistently were negative for EdU, suggesting that a proportion of the Prox1+ cells were post-mitotic. All Prox1, EdU double-positive cells were negative for p27Kip1 (Fig. 4B, grey arrow head), consistent with that these cells have an active cell cycle.

### Cyclin B1- cdk1 is Expressed during Mitotic Entry of Basal HPCs

Our results suggest that the final cell cycle of Lim1+ HPCs is heterogenic with regard to when the cells exit the cell cycle and we propose the existence of three behaviours. Cells with behaviour “two” have an S-phase but do not divide and remain as replicated cells in G2-phase and migrate to the HC layer. The existence of these cells is supported by the FACS and FISH results. Cells with behaviour “three” have an S-phase and basal mitosis before migrating to the HC layer. The S-phase and the basal mitosis have been described previously [10] and can be visualized by EdU incorporation and PH3 immunoreactivity. We show that all cells with behaviour “three” have their S-phases late during the period of Lim1+ HC generation (st26–st31), just prior to their mitosis. The transition from G2 to M-phase is regulated by and dependent on the accumulation of active cyclin B1-Cdk1 in the nucleus [40]. We analysed the intracellular distribution of cyclin B1 in the basal Lim1+ HPCs by using a cyclin B1-GFP vector. The cyclin B1-GFP expression is driven from a human *CCNB1* promoter that gives a normal cell cycle profile. The cellular localization and cyclic degradation of cyclin B1 in chicken cells was confirmed by transfecting chicken DF1 fibroblasts in culture and green fluorescence was translocated from the cytoplasm into the nucleus at M-phase as monitored by PH3 immunoreactivity (Supporting Fig. S5A–C).

The vector was introduced by *in ovo* electroporation of st22 retina and analysed at st25. This stage is before the first basal HPC Lim1+ mitoses and thus before the occurrence of behaviour “three” HPCs. The cyclin B1-GFP fluorescence was below detection in the majority of the interphase cells, which was the expected pattern. Cyclin B1-GFP was localized to the nuclei of cells undergoing apical mitoses as identified by PH3 immunoreactivity on apical side of the retina (Fig. 4C). Cyclin B1 was not seen in the st25 HPCs that had accumulated on the basal side of the retina, as shown by the absence of GFP fluorescence in basal Lim1+ cells (Fig. 4C). When the experiment was repeated and analysed at st27, basal cyclin B1-GFP fluorescent cells were also Lim1, PH3 double-positive (Fig. 4D). Overall, cells with nuclear cyclin B1-GFP were PH3 positive (Fig. 4C–D). This shows that the cyclin B1 level was below detection in basal st25 HPCs (hypothesised to be cells with behaviour “one” (G0) or “two” (replicated state/G2 (G0)) at this stage) but at st27 the level of cyclin B1-GFP is detectable in PH3, Lim1 double-positive cells on the basal side of the retina (Fig. 4D). These observations support our hypothesis that the basal Lim1+ HPCs have different behaviours during their final cell cycle.

### Cdc25C in PH3 Positive HPCs

The activity of the cyclin B1-Cdk1 complex and the progression into M-phase is regulated by the cdc25C phosphatase [40]–[42]. We analysed cdc25C immunoreactivity in Lim1+ HPCs cells in st29 retinas. PH3, Lim1 double-positive cells (Fig. 4E) and PH3+, Lim1 negative cells were cdc25C positive. Lim1+, PH3 negative cells on the basal side of the retina were all cdc25C negative (Fig. 4E, 100 cells inspected). These results were consistent with those of the cyclin B1-GFP analysis, in that neither cyclin B1 nor cdc25C were detected in HPCs before late G2-phase and M-phase (Fig. 4C–E). Among the basal HPCs, only cells with behaviour “three” enter M-phase and were the only ones to be cyclin B1-GFP and cdc25C-positive. Lim1, cdc25C double-positive cells were not seen at st25. The low cyclin B1 and cdc25C levels during the early G2-phase and the increase in M-phase (PH3+ cells) are indicative of a normal G2 to M-phase transition and entry into M-phase by cells with behaviour “three”.

## Discussion

**Figure 5:**
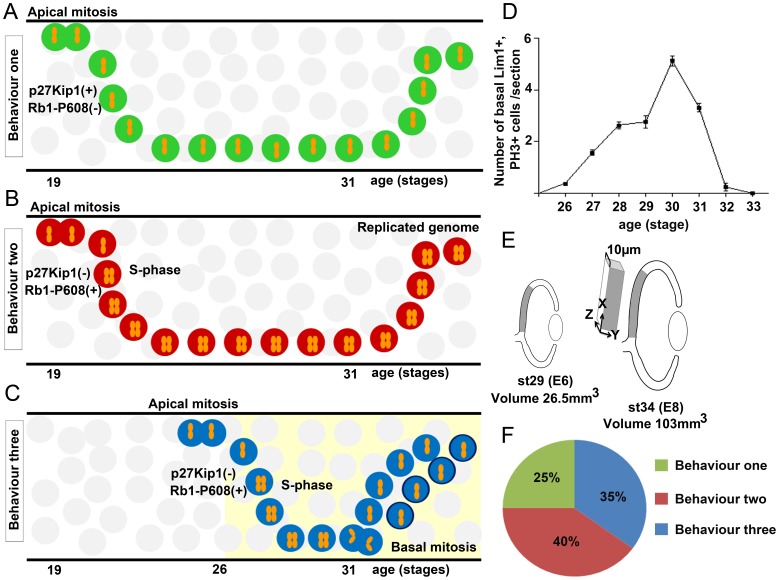
The three proposed final cell cycle behaviours of Lim1+ HPCs and an estimation of the contribution by each HPC-behaviour to the Lim1+ HCs. The Lim1+ HCs have been categorised, based on their final cell cycle, into three different behaviours. (A) Cells with behaviour “one” undergo an apical mitosis and migrate bi-directionally as post-mitotic cells. These cells express p27Kip1. (B) Cells with behaviour “two” initiate S-phase while migrating to the basal side. During their S-phase they express phosphorylated Rb1-S608. These cells do not enter M-phase and will reside in the HC layer with a replicated genome and are heteroploid. (C) Cells with behaviour “three” have an S-phase while migrating to the basal side followed by a non-apical, basal mitosis before migration to the prospective HC layer (st26–31, period indicated by yellow shaded background). (D) The number of Lim1+ basal mitoses per 10 µm section from st26 to st33. Integration of the curve gave an estimation of the total number of mitoses during the period st26–st33 with in total 130 Lim1+ basal mitoses/10 µm section. The length of the late G2/M-phase (PH3 staining) during the investigated stages was assumed to be approximately 1 h [54] and is shorter than the sampling frequency (12 h/st). The risk of counting a cell with a basal mitosis twice is very little and no compensation is required. 130 Lim1+ basal mitoses were assumed to give rise to 260 Lim1+ cells in the mature retina (per 10 µm section). (E) Schematic diagram of the retina with the region used for counting (grey area). As the eye grows during development the central part of the retina expands. By counting a larger central region, we compensated for the growth of the retina along the length of the retinal section. However, as the retinas were sectioned in 10 µm sections independent of age, the corresponding growth related to the thickness of the section (Z-dimension, section thickness) had to be compensated for. The volume of the eye at st29 is 26.5 mm^3^ and at st34 103 mm^3^ [55]. The increase in volume (3.9 times) allowed for calculation of an estimated increase in the Z-dimension (3√3.9 = 1.57). By counting all Lim1+ cells (471±63 cells n = 4 in the HC layer/10 µm) in the central region of st34 retinas and compensate for the growth in the Z-dimension gave approximately 740 Lim1+ HC cells per “compensated section”. Thus, the fraction of Lim1+ cells that are generated by behaviour “three” HPCs is approximately 35%. (F) Contributions by the three HPC-behaviours to the Lim1+ HC population. The Lim1+ HCs generated by behaviour “two” determined by the FACS analysis is based on the assumption that the fraction of Lim1+ cells and Prox1+ cells with increased DNA content is similar. HCs generated from behaviour “one” HPCs corresponds to the remaining Lim1+ cells when behaviour “two “and “three” HPCs are subtracted. st; developmental stage according to Hamburger and Hamilton.

In this work we have studied the final cell cycle of Lim1+ HPCs in the chicken retina. We focused on how the cells exit the cell cycle. The results show that Lim1+ HPCs are heterogenic with regard to when and during which phase the cell cycle is exited. We used markers for S- and G2/M-phase in combination with markers for cell cycle regulators Rb1, cyclin B1, cdc25C and p27Kip1 to characterise the final HC cell cycle and Lim1+ HPCs exhibit three different behaviours during their final cell cycle (Fig. 5, Table 1). Cells with behaviour “one”, exit the cell cycle after INM and an apical mitosis, and then migrate as post-mitotic Lim1+ cells to the basal side of the retina. Cells with behaviour “two” enter S-phase while migrating to the basal side. The S-phase of these cells is not followed by mitosis and these heteroploid cells remain with a replicated genome. Cells with the third type of behaviour, undergo S-phase while migrating to the basal side followed by a delayed non-apical mitosis on the basal side of the retina. We suggest that this third behaviour is equivalent to the delayed terminal mitoses that occur during the formation of the cortex.

**Table 1:** The three cell cycle behaviours of Lim1+ HPCs.

Behaviour	Features
“One”	Bi-directional migration without a delayed final cell cycle
“Two”	S-phase while migrating to the basal side, not followed by mitosis. These cells remain in the HC layer with a replicated genome.
“Three”	S-phase while migrating to the basal side, followed by a non-apical (basal) mitosis before migration to the prospective HC layer.

The results show that all Lim1+ HPCs with a basal mitosis, which we categorise as cells with behaviour “three”, have had S-phase within 6 h before the mitosis and all mitoses occurred after st26. By counting all basal Lim1+ HPCs mitoses and the total number of post-mitotic Lim1+ HCs, we estimated the proportion of HCs that was generated by behaviour “three” HPCs to be approximately 35% (Fig. 5D–F). The EdU data from st24 show that many Lim1+ cells undergo S-phase before st26 and that Lim1+ cells accumulate on the basal side of the retina before the first basal mitoses take place. Such cells were categorised as behaviour “two” and the FACS analysis indicated that at most 40% the Prox1+ HCs were generated from behaviour “two” HPCs. The Prox1+ HCs consist of equal parts of Lim1+ and Isl1+ HCs [11], [17]. Because staining with the Lim1 antibody did not work in combination with the FACS analysis and Isl1 is expressed mainly in non-HC retinal cell types, we could not experimentally determine the proportion of Lim1+ HCs with increased DNA content. Therefore we assumed a proportion based on that the Lim1 and Isl1 HC subpopulations were similar with respect to their final cell cycle. The assumption find support in that the Isl1+ HPCs are formed by non-apical mitoses (behaviour “three”) to a similar degree as the Lim1+ HPCs [11] and display a behaviour “one”-like accumulation on the basal side of the retina. Thus, we assumed that 40% of the Lim1+ HC were generated by behaviour “two” HPCs and the rest (25%) should then be generated from HPCs with behaviour “one” (Fig. 5F). The presence of behaviour “two” cells was supported by the nucleus volume analysis (Fig. 3C and D) and indirectly by the chromosome FISH analysis. However, the frequency of the cells varied between the methods. About 2% of the HCs was identified by the chromosome FISH analysis, 40% by the FACS and 46% was identified by the nuclear volume analysis. This discrepancy may be due to that sister chromatides are associated by cohesins during and after S-phase and FISH double spots are therefore less frequent [43]. Another, explanation may be that the genome in behaviour “two” HPCs is partially replicated and this would also explain that the frequency of cells with two Z-chromatides is lower. Moreover, we do not exclude that the FACS analysis result includes a fraction of cell doublets, although care was specifically taken to exclude all aggregates. The three HPC behaviours overlap and vary over time and therefore an exact determination of their contributions to the final number of HCs, may be elusive.

Neurons with poly- or aneuploidy in adult tissue have been suggested in cat cerebellar cells [44], [45] and human Purkinje cells [46]. More recent findings are chromosomal variations in developing and adult cortical neurons [47], [48] and tetraploidy in chicken retinal ganglion cells described by Morillo and colleagues [32]. We looked for cells with double-spots in the ganglion cell layer using the FISH analysis and found 2 cells with double spots in 200 inspected cells in the ganglion cell layer, which is less than the described frequency of 6% [32]. This indicates that the FISH method underestimates the number of cells with behaviour “two”. The FACS analysis showed that a fraction of the Prox1 negative cells had increased DNA content and ganglion cells with increased DNA content may fall into that category. Reversely, Isl1+ HCs with increased DNA content may have contributed to the fraction of heteroploid Isl1+ cells, described by Morillo et al [32].

The heterogeneity in HC formation has also been seen when comparing species. Mouse and rat HCs migrate to, and accumulate on the basal side of the retina as post-mitotic Lim1+ cells before settling in the HC layer [49] and that corresponds to behaviour “one”. Zebrafish HCs divide non-apically in the HC layer [12] and those mitoses mostly resemble the mitosis seen by behaviour “three” cells in the chicken. We did not find PH3+ cells in the HC layer (up to st44) indicating that there are no non-apical mitoses in the chicken HC layer. However, we cannot exclude that cells undergo a delayed mitosis after hatch.

We studied Rb1, cyclin 1B, cdc25C and p27Kip1 with focus on the HPCs (Lim1+ or Prox1+) and in relation to the three proposed final cell cycle behaviours. Rb1 regulates S-phase entry [50] and at st24 Lim1, EdU double-positive (behaviour “two”) cells were Rb1-P608+ as expected (Fig. 4A). The st24 Lim1+, EdU negative cells were negative for Rb1-P608 and represent cells with behaviour “one”. Behaviour “one” cells should be post-mitotic and when the expression of p27Kip1 was analysed, only Prox1+, EdU negative cells were p27Kip1+, which is consistent with a post-mitotic cell (Fig. 4B). p27Kip1-expression promotes cell cycle exit and loss of one or both alleles of p27Kip1 leads to extra rounds of cell division during development [35]. The expression of Rb1-P608 and p27Kip1 in st24 Lim1+ or Prox1+ cells gives support to the hypothesised final cell cycle in both behaviours “one” and “two”. The transition of the cell cycle phases G2 to M-phase is characterized by an increase and activation of cyclin B1, Cdk1 and cdc25C [51], [52]. We neither detected cyclin B1 nor cdc25C in the basal PH3 negative HPCs (Fig. 4C and E), whereas cyclin B1 and cdc25C were seen in the Lim1, PH3 double-positive HPCs (Fig. 4D and E) indicating that neither cyclin B1 nor cdc25C is present in Lim1+ or Prox1+ cells with behaviour “one” or “two”.

The HCs and their final cell cycle are of particular interest since it has been shown that the HCs are potential “cells of origin” for retinoblastoma. Mouse HCs in the retina of Rb-family knock-out mice defy signals to become post-mitotic and instead continue to proliferate and develop a malignant tumour [19]. Furthermore, interfering with microtubule polymerization causes Rb1 expressing cells to arrest as tetra- or aneuploid cells whereas Rb1 deficient cells accumulate with higher ploidy as they bypass the mitotic block [53]. Our results indicate that the final cell cycle by chicken Lim1+ HCs is heterogenic and we propose that at least three different final cell cycle exit behaviours can be distinguished (Fig. 5). The final cell cycle heterogeneity may present a cellular context that is more permissive to tumour development when Rb1 is mutated.

## Supporting Information

Figure S1 **Length of the EdU pulse after eye or yolk sac injections.**
(PDF)

Figure S2 **Fraction of the Prox1+ cells that are located in the horizontal cell layer.**
(PDF)

Figure S3 **Quick-time movie of an animated rotation of the 3D reconstruction of a cell having two FISH-signals.** Animation of a 3D reconstruction of Lim1+ HC nuclei in the HC layer of a st42 female retina. The nuclei are rotated to display the FISH-signal in relation to the nuclei. The 3D reconstruction and animation were based on confocal sections through the entire HC layer in Imaris (Bitplan software). The movie shows Lim1+ (green) HC nuclei with fluorescence FISH-signals (red spots) in the horizontal cell layer of a st42 retina. The FISH Z-chromosome BAC probe generates a single spot-signal in normal diploid nuclei. The Lim1+ cell located in the middle has two red spots indicating a replicated genome.
(MPG)

Figure S4 **Evaluation of the Z-BAC probe by metaphase chromosome FISH analysis.**
(PDF)

Figure S5 **Control experiments to test the cyclin B1-GFP in cultured cells.**
(PDF)
