# Supplementary material for: Heterogenic Final Cell Cycle by Chicken Retinal Lim1 Horizontal Progenitor Cells Leads to Heteroploid Cells with a Remaining Replicated Genome
Source: PLoS One. 2013 Mar 19;8(3):e59133. doi: 10.1371/journal.pone.0059133 (PMC3602602; doi:10.1371/journal.pone.0059133)
Supplement: Figure S1 — Length of the EdU pulse after eye or yolk sac injections. (PDF) [file pone.0059133.s001.pdf]

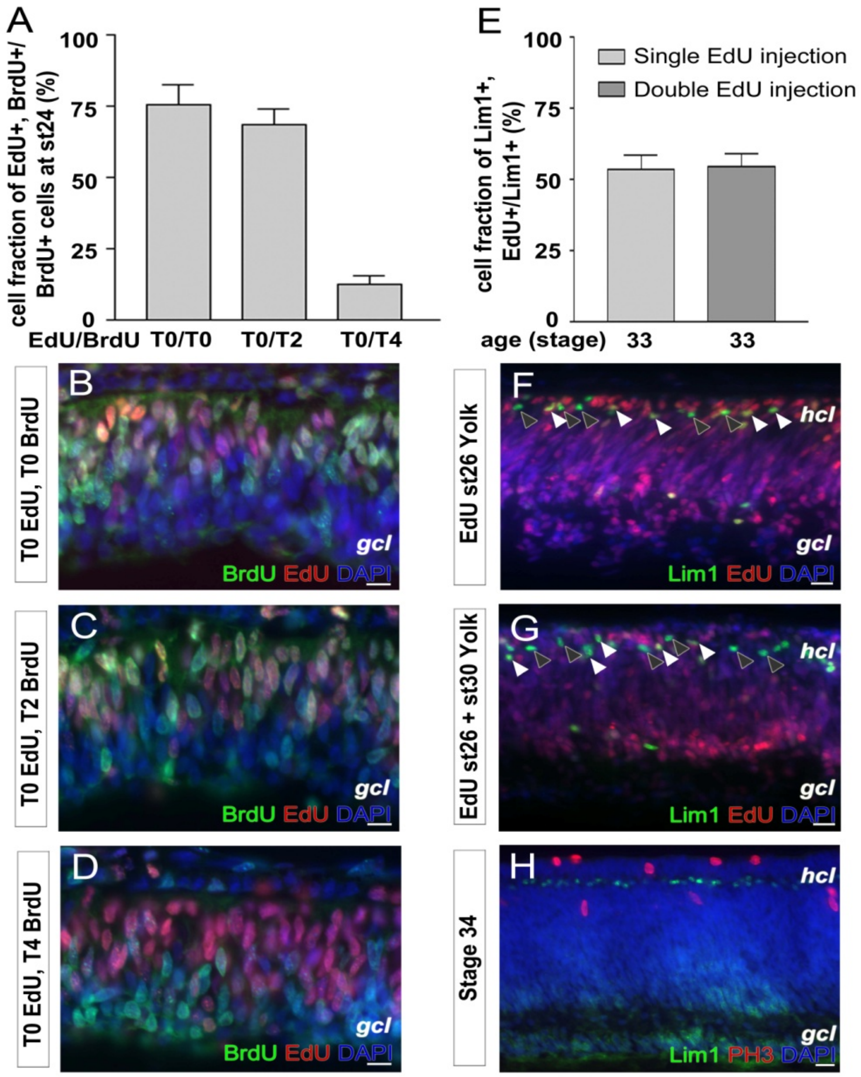

Supplemental figure S1. Length of the EdU pulse after eye or yolk sac injections.

(A) Bargraphs with the fraction of BrdU+ cells at st24 that have incorporated EdU after single eye injections with the intervals 0, 2 and 4 h. (B) EdU and BrdU immunolabelling after simultaneous (T0) EdU and BrdU single injections. (C) BrdU 2 h (T2) after an EdU injection and (D) BrdU 4 h (T4) after an EdU injection in st24 retinas. (E) Bargraph showing the fraction of Lim1+ cells at st33 that have incorporated EdU after a single yolk injection compare to repeated (double) injections (st26 and st30) into the yolk sac. (F) Lim1 and EdU immunolabelling in st33 retinas after a single EdU yolk sac injection at st26. (G) Lim1 and EdU immunolabelling in st33 retinas after two repeated injections. (H) Fluorescence micrographs of st34 retina with Lim1 and PH3. White arrow head; double-positive cell, gray arrow head; single-positive cell. gcl; ganglion cell layer, hcl; horizontal cell layer, st; Hamburger and Hamilton stages. Scale bar is 10µm.
