# Supplementary material for: Heterogenic Final Cell Cycle by Chicken Retinal Lim1 Horizontal Progenitor Cells Leads to Heteroploid Cells with a Remaining Replicated Genome
Source: PLoS One. 2013 Mar 19;8(3):e59133. doi: 10.1371/journal.pone.0059133 (PMC3602602; doi:10.1371/journal.pone.0059133)
Supplement: Figure S2 — Fraction of the Prox1+ cells that are located in the horizontal cell layer. (PDF) [file pone.0059133.s002.pdf]

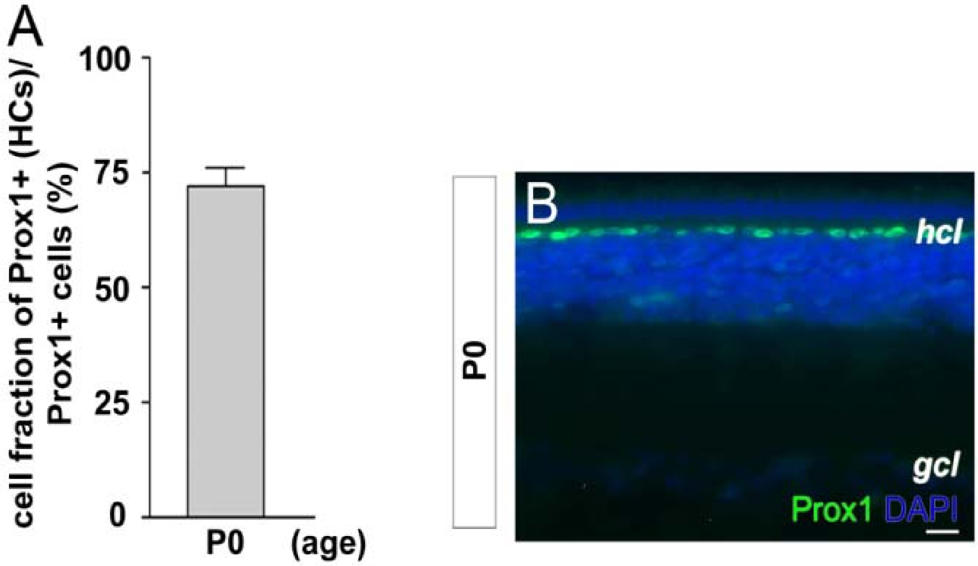

Supplemental figure S2. Fraction of Prox1+ cells that are HCs

(A) Bargraph with the fraction of Prox1+ cells located in the HC layer compare with the total number of Prox1+ cells at P0. (B) Fluorescence micrographs with Prox1 on P0 retina.gcl; ganglion cell layer, hcl; horizontal cell layer, P0 = post hatched. Scale bar is 10μm.
