# Supplementary material for: Heterogenic Final Cell Cycle by Chicken Retinal Lim1 Horizontal Progenitor Cells Leads to Heteroploid Cells with a Remaining Replicated Genome
Source: PLoS One. 2013 Mar 19;8(3):e59133. doi: 10.1371/journal.pone.0059133 (PMC3602602; doi:10.1371/journal.pone.0059133)
Supplement: Figure S4 — Evaluation of the Z-BAC probe by metaphase chromosome FISH analysis. (PDF) [file pone.0059133.s004.pdf]

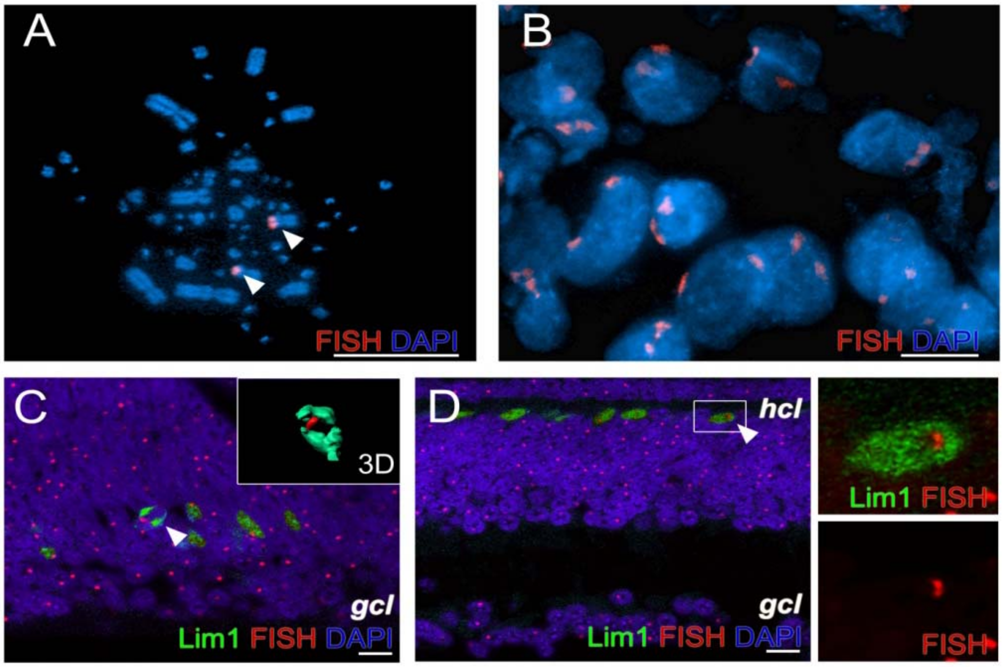

Supplemental figure S4. Evaluation of the Z-BAC probe by metaphase chromosome FISH analysis.

(A) Metaphase chromosome FISH analysis of a male DF1 (embryonic fibroblast) cell showed that the probe specifically hybridized to the tip of one chromosome pair with a relative size that is corresponding to the Z-chromosome. White arrow head: FISH signal. (B) FISH analysis of interphase nuclei of cultured male chick DF1 cells. Note that the fluorescent signal is located in the periphery of the nucleus. (C) Fluorescence micrograph of a confocal image section in the far periphery of st33 female chicken retina and in the region with basal HC mitoses. The FISH analysis produces one red fluorescence signal (spot) per diploid female cell. White arrowhead points at an elongated spot in a Lim1+ cell that is approaching mitosis. The insert is a 3D reconstruction based on confocal sections through the entire Lim1+ nucleus with the Z-probe-signal being exposed in the image (Imaris software). (D) Fluorescence micrograph of a Lim1+ cells with an elongated fluorescent spot. White arrow head; cell with elongated-spot. gcl; ganglion cell layer, hcl; horizontal cell layer. Scale bar is 10µm.
