# Supplementary material for: Heterogenic Final Cell Cycle by Chicken Retinal Lim1 Horizontal Progenitor Cells Leads to Heteroploid Cells with a Remaining Replicated Genome
Source: PLoS One. 2013 Mar 19;8(3):e59133. doi: 10.1371/journal.pone.0059133 (PMC3602602; doi:10.1371/journal.pone.0059133)
Supplement: Figure S5 — Control experiments to test the cyclin B1-GFP in cultured cells. (PDF) [file pone.0059133.s005.pdf]

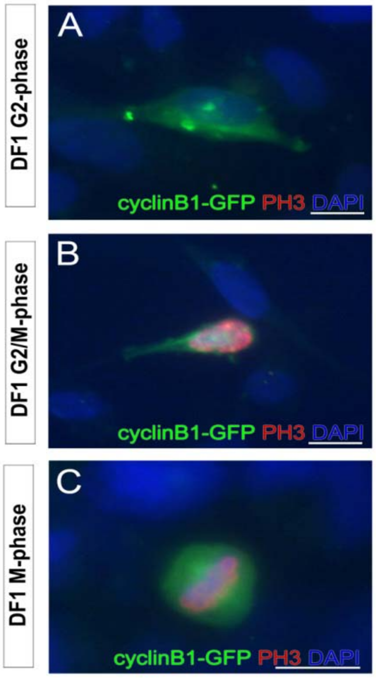

<u>Supplemental figure S5. Control experiments of the cyclin B1-GFP in cultured cells.</u>

(A-C) DF1 cells (chicken embryonic fibroblasts) were transfected with cyclin B1-GFP vector and labelled for phospho-histone H3 (PH3). (A) Fluorescence micrographs showing a DF1 cell in G2-phase with fluorescence in cytoplasm, (B), G2/M-phase: PH3 labelled with fluorescence in nucleus (C) and M-phase. Scale bar is 10µm.
